# Urokinase-type plasminogen activator supports liver repair independent of its cellular receptor

**DOI:** 10.1186/1471-230X-6-40

**Published:** 2006-11-29

**Authors:** Kumar Shanmukhappa, Gregg E Sabla, Jay L Degen, Jorge A Bezerra

**Affiliations:** 1 Division of Pediatric Gastroenterology, Hepatology, and Nutrition. Cincinnati Children's Hospital Medical Center and the Department of Pediatrics of the University of Cincinnati College of Medicine, Cincinnati, Ohio, USA; 2 Division of Developmental Biology, Cincinnati Children's Hospital Medical Center and the Department of Pediatrics of the University of Cincinnati College of Medicine, Cincinnati, Ohio, USA

## Abstract

**Background:**

The urokinase-type (uPA) and tissue-type (tPA) plasminogen activators regulate liver matrix remodelling through the conversion of plasminogen (Plg) to the active protease plasmin. Based on the efficient activation of plasminogen when uPA is bound to its receptor (uPAR) and on the role of uPA in plasmin-mediated liver repair, we hypothesized that uPA requires uPAR for efficient liver repair.

**Methods:**

To test this hypothesis, we administered one dose of carbon tetrachloride (CCl_4_) to mice with single or combined deficiencies of uPA, uPAR and tPA, and examined hepatic morphology, cellular proliferation, fibrin clearance, and hepatic proteolysis 2–14 days later.

**Results:**

Absence of uPAR alone or the combined absence of uPAR and tPA had no impact on the resolution of centrilobular injury, but the loss of receptor-free uPA significantly impaired the clearance of necrotic hepatocytes up to 14 days after CCl_4_. In response to the injury, hepatocyte proliferation was normal in mice of all genotypes, except for uPAR-deficient (uPAR°) mice, which had a reproducible but mild decrease by 33% at day 2, with an appropriate restoration of liver mass by 7 days similar to experimental controls. Immunostaining and zymographic analysis demonstrated that uPA alone promoted fibrin clearance from centrilobular regions and efficiently activated plasminogen.

**Conclusion:**

uPA activates plasminogen and promotes liver matrix proteolysis during repair via a process that neither requires its receptor uPAR nor requires a contribution from its functional counterpart tPA.

## Background

Tissue repair and remodelling in response to injury require well-coordinated cellular proliferation in synchrony with reorganization of the extracellular matrix. After a toxic injury resulting in focal hepatic necrosis, liver cells undergo proliferation events to recover the original hepatic mass in a time-restricted fashion [1-3]. This proliferative response must be matched by the proteolytic clearance of necrotic cells and matrix reorganization to restore the lobular architecture [4-6]. While the proliferative response is regulated by the expression of cytokines, growth factors and transcription factors [7-9], debris clearance and extracellular matrix remodelling are driven in part by tissue-derived and circulating proteases. Based on the observation that liver repair is severely compromised in mice lacking plasminogen (Plg), the Plg family of proteases emerged as key mediators of matrix proteolysis/remodelling following an injury. The hepatic proliferative response is not impeded in Plg-deficient mice following a single administration of hepatotoxin, but the clearance of necrotic foci and the restoration of normal lobular organization are severely impaired within the liver of Plg-deficient mice relative to control animals [6,10,11].

In order to exhibit proteolytic activity with macromolecular substrates, Plg must be proteolytically converted to the two-chain serine protease plasmin by urokinase-type (uPA) or tissue-type (tPA) plasminogen activator. uPA has been proposed to play an important role in liver regeneration based on a decrease in the proliferative response of hepatocytes following partial hepatectomy [12]. However in the model of liver repair due to toxic insult, we found that loss of uPA did not result in decreased proliferation of hepatocytes [13]. The reason for this discrepancy in the role of uPA regulating hepatocyte proliferation may be due to differences in molecular pathways regulating the hepatic response to a physical injury (as in partial hepatectomy) and a toxic insult (CCl_4 _injury), as well as injuries induced by other experimental models such as the administration of anti-Jo2 antibody [14]. In this experimental context, the loss of uPA resulted in a moderate defect in liver repair despite normal hepatic proliferation and the combined loss of uPA and tPA led to a more profound defect in liver repair akin to the findings in Plg-deficient mice [13]. Exuberant transgene-mediated hepatic expression of uPA failed to correct the reparative defect observed within the liver of Plg-deficient mice [15], suggesting that plasmin(ogen) is central to hepatic remodelling after acute toxic injury. However, it remains to be determined whether binding of uPA to its receptor (uPAR) on the surface of either hepatocytes or other cells is important for liver repair by localizing Plg activation to the immediate pericellular microenvironment.

Binding of uPA to its cellular receptor uPAR is important for the efficient activation of plasminogen at the cellular surface in several in vivo and in vitro systems [16-21]. In addition to supporting pericellular zymogen activation, the formation of the uPA-uPAR complex on the cell surface is known to influence cell adhesion, migration, and chemotactic properties [17-20,22]. Based on these findings, we hypothesized that binding of uPA to uPAR is required for efficient liver repair. To address this question, we investigated the hepatic reparative response in mice with single or combined deficiencies in uPA, uPAR and tPA. We report that uPA-mediated plasminogen activation is important for the timely repair of the hepatic tissue following an acute injury, but efficient liver repair can be achieved in the absence of uPAR and in the absence of any benefit of tPA-mediated plasminogen activation.

## Methods

### Gene-targeted mice

Mice with individual disruption of the genes encoding uPA (uPA°), uPAR (uPAR°), and tPA (tPA°) were of mixed genetic background 129/C57BL/6, and were genotyped by PCR using tail DNA and specific primers as described previously [13,23]. Mice with the combined loss of uPAR and tPA (uPAR°/tPA°) were also included in the experimental design to generate a biological system displaying soluble uPA as the only means to activate Plg [23,24] ; non-targeted wild-type (WT) littermates served as controls. All the experimental challenges were performed in 2- to 6-month old littermates housed in standard facilities and fed food and water ad libitum. Animal protocols were approved by the Institutional Animal Care and Use Committee of the Cincinnati Children's Research Foundation (Cincinnati, OH, USA).

### Liver injury

Gene-targeted mice and control littermates were challenged with a single intraperitoneal injection of CCl_4 _(Aldrich Chemical Inc., Milwaukee, WI) at a dose of 12.5 μl CCl_4 _per 25-gram body weight delivered in a 25% solution in corn oil. Mice were examined daily and sacrificed at days 2, 7, and 14 after CCl_4 _as described previously [6,11]. In brief, the mice were weighed, sacrificed, and blood was collected from the inferior vena cava at the time of sacrifice. Livers were excised, embedded in paraffin, sectioned, and stained with hematoxylin and eosin for histological analysis. Biochemical markers of liver function and injury were determined in plasma by automated enzymatic assay using Vistros Chemistry Systems 950 (Johnson and Johnson, Rochester, NY) [25].

### Zymography to determine Plg activation

Liver protein lysates were isolated by homogenization in solubilization buffer containing 1% Nonidet-P40, 0.5% deoxycholate, 0.1% Sodium Dodecyl Sulfate, and 10% Protease inhibitor cocktail (Sigma Chemical Co., St. Louis, MO) as described previously [13]. After homogenization, the soluble fraction was collected from the supernatant by centrifugation at 12,000 *g *for 15 min. Protein concentration was determined using the Bradford method based Bio-Rad assay (Bio-Rad Laboratory Inc., Hercules, CA). For zymography, 50 μg of total protein was assayed using 12.5% polyacrylamide gel cast containing 0.4% nonfat dry milk and 20 μg/ml Plg, as described previously [13]. Briefly, gels were washed twice for 30 min in 2.5% Triton X-100 after electrophoresis and then incubated for 16 hr at 37°C in 0.1 M glycine (pH 8.0). Caesinolytic areas were determined by Coomasie blue staining.

### Fibrinogen staining

To investigate the deposition of fibrin(ogen) in the hepatic lobule, immunohistochemistry was performed on liver sections using a rabbit anti-fibrin(ogen) antiserum followed by detection with the Vectastain ABC-AP staining system (Vector Laboratories, Burlingame, CA) and Fast Red TR/naphthol AS-MX (Sigma Chemicals, St. Louis, MO) as described previously [11].

### Hepatocyte proliferation

The proliferative response of hepatocytes after CCl_4 _injection was measured by incorporation of bromodeoxyuridine (BrdU), which was administered intraperitoneally to all mice 2 hr before sacrifice as described previously [25]. BrdU-labelled hepatocytes were identified on 4 μm sections of paraffin-embedded liver samples according to manufacturer's instructions (Cell proliferation kit, Amersham Life Science, Arlington Heights, IL) as described previously [13]. For each sample, the hepatocyte-labelling index (percent of hepatocytes incorporating BrdU) was calculated by counting BrdU-labelled and unlabeled hepatocyte nuclei in 10 high-power fields (~100 hepatocyte nuclei/field) by an investigator unaware of the animal genotype. Hepatocyte proliferation was expressed as the mean +/- standard deviation (SD) for all mice in each group (n = 3–6 mice/group).

### Purification of hepatocyte growth factor

Cytosolic hepatocyte growth factor (HGF) was partially purified from livers as described previously [26]. Briefly, livers were homogenized in 4 volumes of ice-cold lysis buffer {50 mM Tris-HCl (8.5), 0.15 M NaCl, 10 mM EDTA, 100 μM Nafamostat mesilate and 1 mM PMSF}, followed by removal of cellular debris by centrifugation at 25,000 *g *for 20 min and 105,000 *g *for 60 min at 4°C. Supernatant containing soluble proteins was supplemented with 0.1% CHAPS and passed through S-Sepharose column. Eluate was concentrated by ultrafiltration using Centricon 30 (Amicon) following the manufacturer's instructions, electrophoresed on 10% SDS-PAGE under reducing conditions, transferred to nitrocellulose membrane, incubated with goat anti-mouse HGF antibody (Santa Cruz Biotechnology Inc, Santa Cruz, CA), AP-conjugated rabbit anti-goat antibody (Vector Laboratories, Burlingame, CA), and the specific signals of single and heavy chains were detected with enhanced chemiluminescence (Amersham Biosciences, Piscataway, NJ).

### Quantification of CD11b cells in the regenerating liver

To isolate hepatic mononuclear cells, peripheral blood was flushed out from the liver by infusion of PBS through the portal vein, followed by excision and mincing of the liver, passing the cell suspension through 40 μm nylon strainer (BD Biosciences, Franklin Lakes, NJ, USA) in RPMI Medium 1640 supplemented with 4% fetal calf serum (Life Technologies Inc., Grand Island, NY, USA) and centrifugation at 75 *g *for 2 min to remove cellular debris as described previously [27]. The cell suspension was treated with red blood cell lysis buffer (0.15 M NH_4_Cl, 10 mM KHCO_3_, and 0.1 mM Na_2_EDTA at pH 7.2), centrifuged at 270 *g *for 10 min, resuspended in PBS containing 4% fetal calf serum, and labelled with anti-mouse Fc II/III receptor mAb (to block non-specific binding) and FITC-conjugated anti-mouse CD11b mAbs (BD Biosciences, San Jose, California, USA), and analyzed in a FACSCalibur dual-laser flow cytometer as described previously [27,28].

### Statistical analysis

Statistical analysis was performed with one-way ANOVA to assess for intergroup and intragroup differences and by unpaired student's t-test for two-group comparisons, with a significance level set at P < 0.05.

## Results

### Outcome of liver repair in uPAR° mice

**Figure 1:**
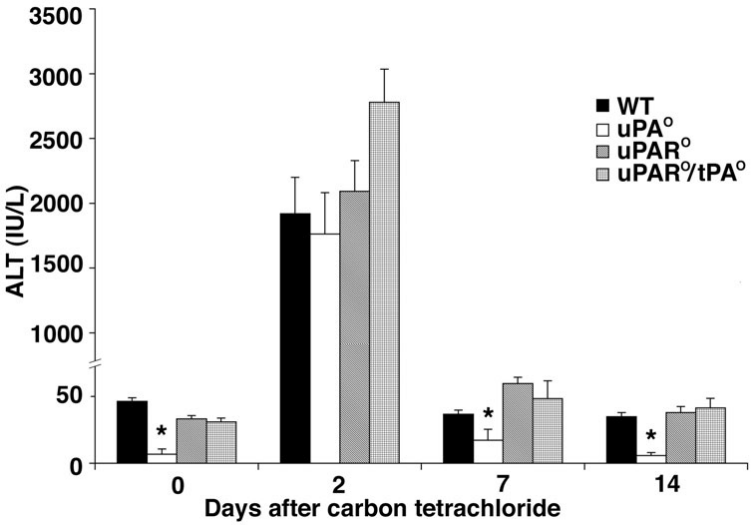
**Levels of serum alanine aminotransferase (ALT) after CCl **_**4 **_. Serum levels of ALT peak 2 days following CCl_4 _administration in mice of all genotypes. Thereafter, the values return to basal levels within 7 to 14 days in a similar manner in all experimental groups (n = 3–8 at each time point). * P = 0.023 when compared experimental controls.

A single dose of the hepatotoxin CCl_4 _results in an acute necroinflammatory injury to centrilobular hepatocytes due to the conversion of CCl_4 _to the free radical CCl_3 _and other highly reactive species [6,29]. To explore the role of uPAR in uPA-mediated plasminogen activation during liver repair, mice were challenged with a single dose of CCl_4_. In keeping with the development of an acute hepatocellular injury, serum levels of alanine aminotransferase (ALT) increased at 2 days and returned to baseline levels by 7 days, regardless of the availability of uPAR or plasminogen activator (Figure 1). Interestingly, uPA° mice displayed low baseline levels of ALT when compared to mice of other genotypes (P = 0.023), but all baseline levels were within normal limits. While the reason for the low baseline level in uPA° mice is not known, ALT levels increased to similar levels of WT mice at the time of acute injury (2 days). Therefore, deficiency of uPA/uPAR did not affect the initial degree of liver damage or the transient hepatotoxicity induced by CCl_4 _as indicated by ALT levels. Consistent with the rise in serum ALT, visual inspection of livers 2 days after CCl_4 _revealed a similar diffuse pale lacy appearance in all mice regardless of genotype (data not shown). The gross appearance of livers of wild type, uPAR° and uPAR°/tPA° mice normalized within 7 days of CCl_4 _administration. This normal appearance of livers from uPAR°/tPA° mice was the first indication that receptor-free uPA was sufficient for the reparative response. In contrast, livers of mice with the single loss of uPA continued to display a pale lacy appearance at 7 days, with slight improvement at 14 days after CCl_4_.

**Figure 2:**
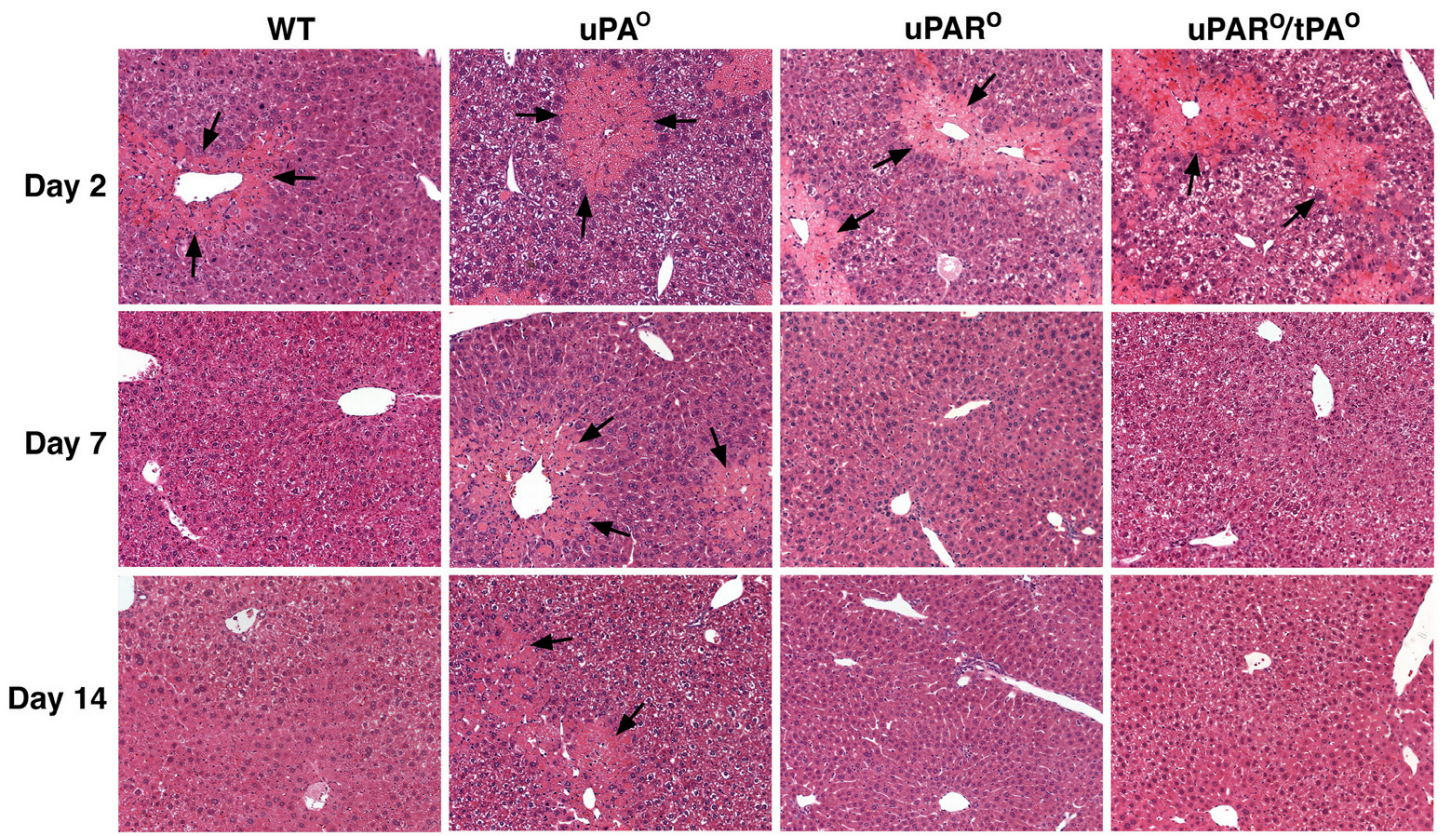
**Impaired resolution of centrilobular injury in mice lacking receptor-free uPA**. Histological liver sections 2 days after CCl_4 _show similar features of centrilobular necrosis (arrows) in mice of all genotypes. While livers from wild type (WT), uPAR°, and uPAR°/tPA° show resolution of the centrilobular injury by day 7, uPA° livers continue to display the injury at 7 and 14 days after CCl_4 _despite the presence of uPAR (Magnification 200×)

To investigate the microscopic basis for the abnormal visual appearance after CCl_4_, we performed histological analysis of paraffin-embedded liver sections of uPAR° mice and experimental controls after CCl_4_. Two days after injury, all livers exhibited widespread liquefaction necrosis in centrilobular hepatocytes regardless of the genotype, with minimal inflammation and intact cellular components in the remainder of the liver lobule (Figure 2). Systematic analysis of liver sections at 7 and 14 days following CCl_4 _showed that the centrilobular injury of uPAR° mice resolved completely by 7 days in a fashion similar to the resolution observed in wild type and uPAR°/tPA° mice (Figure 2). This was in stark contrast to the persistent centrilobular injury in livers of uPA° mice that persisted through 14 days. The persistence of the centrilobular injury in the setting of normalization in the levels of ALT is consistent with a defect in repair, rather than ongoing injury, as described previously [13].

Collectively, these data indicate that the presence of uPA is critical for the efficient resolution of the centrilobular injury; but the loss of uPAR does not appreciably hinder the ability of receptor-free uPA to orchestrate the reparative response of the liver to CCl_4_.

### Lack of uPAR results in a mild, transient decrease in hepatocyte proliferation

**Figure 3:**
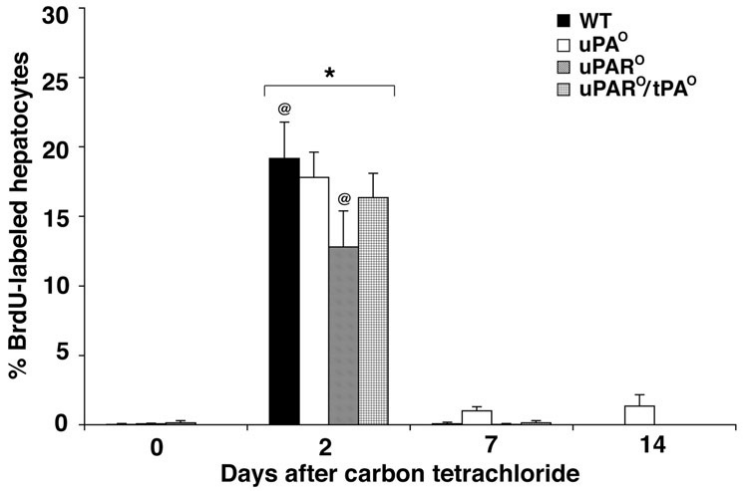
**Transient decrease in proliferation of hepatocytes in mice lacking uPAR**. Quantification of BrdU-labeled hepatocytes shows a rise in BrdU uptake in all experimental groups 2 days after CCl_4_, with a decrease of ~33% in uPAR° livers. The percent of BrdU-labeled hepatocytes return to near baseline levels in all groups. WT = wild type. (*ANOVA P < 0.004; ^@^t-test P < 0.004; N = 3–8 at each time point).

**Figure 4:**
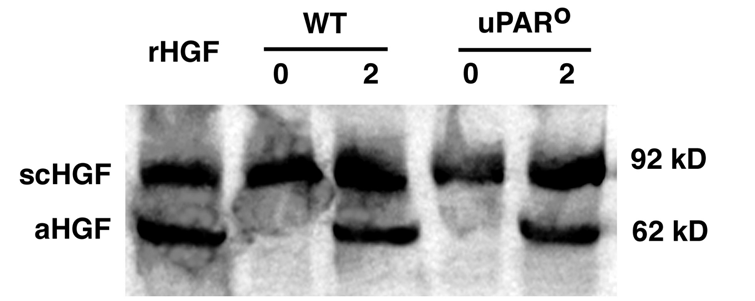
**Normal activation of cell-associated hepatocyte growth factor in uPAR° livers during liver repair**. Western analysis of hepatocyte growth factor (HGF) partially purified from the soluble fraction of liver extracts using an antibody that recognizes the single chain and heavy chain (to demonstrate activation) of HGF shows similar levels of activation in WT and uPAR° livers at baseline (day 0) or at 2 days after CCl_4 _administration. scHGF = single chain HGF; aHGF = activated HGF, rHGF = recombinant HGF.

**Figure 5:**
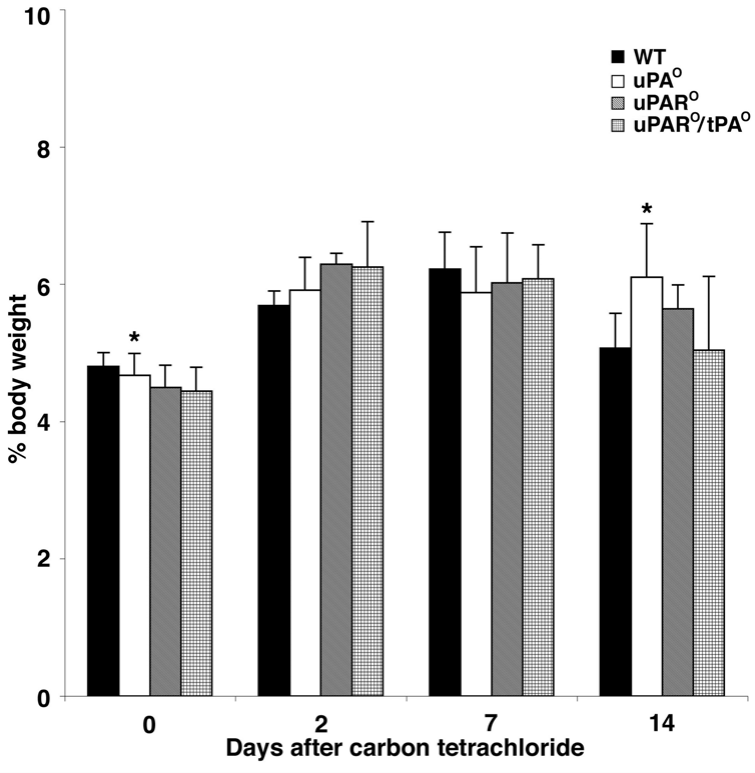
**Maintenance of liver mass following CCl **_**4 **_. Analysis of the liver mass (expressed as the wet liver weight as a percentage of the body weight) shows a mild increase in all genotypes 2 days after CCl_4 _administration, followed by a trend toward baseline levels by 14 days, except for livers of uPA° mice which continue to display a mass that is similar to day 2 (and higher than before CCl_4_, *P < 0.03). WT = wild type.

Based on the pleiotropic role of uPAR as a regulator of cell proliferation, pericellular proteolysis, and cellular differentiation [30,31], we investigated whether the loss of uPAR impairs hepatocyte proliferation after CCl_4 _administration. Using BrdU incorporation by hepatocytes as an indicator of proliferation, we found that hepatocyte proliferation increased substantially in all mice regardless of the genotype 2 days after CCl_4_. Interestingly, the proliferative index was mildly lower in livers of uPAR° mice (one-way ANOVA, P < 0.002), 33.3% below the index in livers from WT mice (Student's t test, P < 0.004; Figure 3). While statistically significant and reproducible in two independent experiments, this difference was modest and did not modify the return to baseline levels of hepatocyte proliferation by 7–14 days. The transient decrease in proliferation did not influence the restoration of the lobular architecture; the persistent centrilobular injury was present only in uPA° mice. Since HGF is a potent mitogen to hepatocytes [32], we reasoned that the transient decrease in hepatocyte proliferation could be due to decreased activation of HGF. To examine this possibility, we used western analysis and found no difference in the expression levels of single-chain or activated forms of cytosolic HGF in uPAR° mice either before CCl_4 _administration or at day 2 following CCl_4 _when compared to WT mice (Figure 4). Because uPAR plays an important role in regulating the migration of macrophages and modulating the expression of cytokines [33-39], we determined the total number of hepatic CD11b-labeled cells in WT and uPAR° mice by flow cytometry 2 days after injury. We found no difference in the number of CD11b-labeled cells (2.4 × 10^6^/livers in WT mice versus 2.2 × 10^6^/livers in uPAR° mice, P = 0.8294, n = 3 for each group). Combined, these data suggest that the transient decrease in hepatocyte proliferation in uPAR° mice is not due to changes in HGF activation or migration of immune cells in these livers. Notably, hepatocyte proliferation did not differ between uPA° and WT mice, but the liver to body weight ratio in uPA° mice showed a clear trend to increase over time (Figure 5); this was similar to the findings previously described in Plg-deficient mice due to the accumulation of fibrin in injured areas [6,13]. Therefore, we next determined whether fibrin clearance is impaired in uPAR° mice.

### Loss of uPAR does not lead to impaired clearance of the provisional fibrin in diseased livers

**Figure 6:**
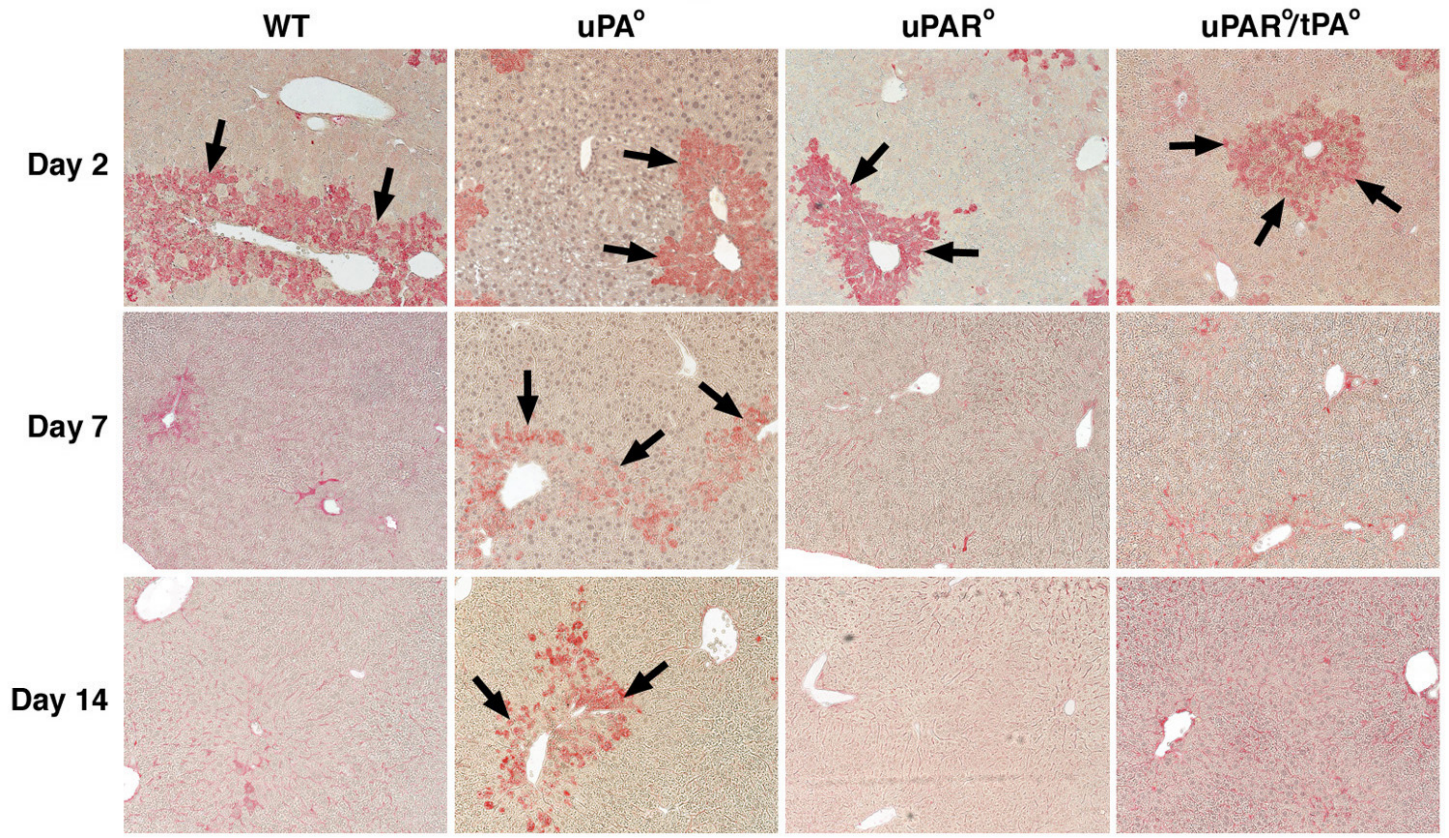
**Receptor-free uPA is required for fibrin clearance**. Detection of fibrin deposition by immunohistochemistry (red stain, arrows) shows uniform staining in centrilobular area in livers of mice of all genotypes 2 days after CCl_4_. The clearance of fibrin coincides with the resolution of the centrilobular injury by day 14 in mice of each genotype, except for uPA° mice. (Magnification 200×)

We investigated whether clearance of fibrin in the hepatic lobule is impaired in uPA°, uPAR° and uPAR°/tPA° mice after CCl_4_. Immunohistochemical analysis of the liver sections 2 days after CCl_4 _showed that fibrin deposition in injured centrilobular regions was a prominent feature throughout the liver lobules in mice of all genotypes (Figure 6). However, this centrilobular fibrin deposition was transient in livers of uPAR° and uPAR°/tPA° mice, which allowed for timely fibrin removal by 7 days similar to WT mice. In mice lacking uPA, fibrin deposition was still evident at 14 days after CCl_4 _administration. These findings imply that uPA promotes the proteolytic clearance of fibrin-rich matrix independent of its high affinity receptor and independent of tPA.

### Total hepatic uPA is similar with and without uPAR

**Figure 7:**
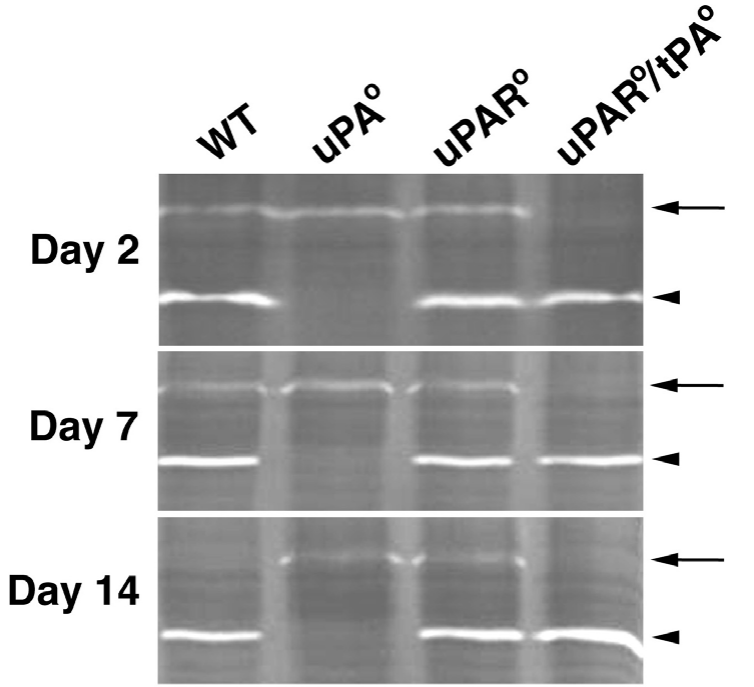
**Hepatic uPA activation occurs in the absence of uPAR**. Activation of uPA in the liver was observed in mice of all genotypes after CCl_4_, except in uPA° mice. The upper band (arrow) depicts tPA while the lower band (arrow head) depicts uPA.

Binding of uPA to uPAR has been shown to increase proteolytic activation in vitro [40]. Thus, to explore whether loss of uPAR alters the level of hepatic uPA, we determined the ability of liver protein extracts to activate Plg. Using zymographic analysis, we found that total liver protein induced activation of Plg at the predicted molecular weight for uPA in hepatic extracts from all genotypes except for livers of uPA° mice (Figure 7). This was evident 2 days after CCl_4_, a time point with similar levels of uPA in wild type, uPAR° and uPAR°/tPA° mice as well as at later time points (7 and 14 days). These results support the hypothesis that the loss of hepatic uPAR does not alter the ability of uPA to induce Plg activation during the reparative response of the liver to an acute injury.

## Discussion

Our data show that among the effectors of plasminogen activation (uPA, tPA and uPAR), uPA alone is able to efficiently promote liver repair. It can do so independently from its receptor and without any added contribution from its functional counterpart, tPA. This was evidenced by the findings that the loss of uPAR did not affect the initial degree or features of the centrilobular injury induced by CCl_4_, or the time required for the liver to restore the normal lobular architecture. Interestingly, there was a mild, transient attenuation in the proliferative response in the absence of uPAR, which did not impact the return to baseline levels of proliferation or restoration of liver mass. Furthermore, the timely clearance of the provisional fibrin matrix in injured centrilobular regions and the levels of uPA in both uPAR° and uPAR°/tPA° livers underscore the non-essential contribution of uPAR and tPA in the liver-specific plasminogen activation/fibrinolysis during liver repair. Collectively, these data demonstrate that while binding of uPA to its receptor may optimize liver cell proliferation and repair, the primary functions of fibrin removal, clearance of necrotic cells, and reorganization of the liver lobule can be efficiently accomplished solely with receptor-free uPA. It is conceivable, if not likely, that both plasminogen activation and plasmin-mediated proteolysis within damaged liver tissue may be less efficient in the absence of uPAR-supported uPA localization. However, if so, then the current data indicate that the residual plasminogen activation and plasmin-mediated proteolysis in uPAR-deficient mice is adequate to support fibrin clearance and hepatic repair that is comparable to wild-type mice. The normal liver repair mediated by uPA in the absence of both tPA and uPAR might argue the case for the existence of undefined compensatory networks or of other receptors for uPA that facilitate plasminogen activation; these molecules, however, are largely undefined. It might also argue for the more likely scenario in which soluble uPA is able to efficiently activate plasminogen and facilitate plasmin-mediated liver repair.

The decrease in liver cell proliferation after an acute toxic injury in uPAR° mice is in keeping with the role of uPAR in promoting cellular proliferation, proteolysis and cellular differentiation in extra-hepatic systems [30,31]. A decrease in hepatocyte proliferation has also been reported following 70% partial hepatectomy in uPA° mice [12]. In these studies, livers of uPA° mice also accumulated fibrin within the lobule of regenerating liver. However, these findings were transient and did not impair the timely restoration of the liver mass or modify the long-term survival of the organism. It is possible that a deficit in the generation of plasmin in the hepatic environment of uPAR° mice may be responsible for the transient depression in hepatocyte proliferation after CCl_4 _based on the role of uPAR and uPA in plasminogen activation [17,41,42]. However, this seems unlikely in view of the normal proliferative response observed in plasminogen-deficient mice following CCl_4 _injury [6]. Alternatively, the decreased proliferation may be due to impaired activation of growth-related signals in uPAR° livers. This scenario is supported by the role of the ligand uPA in the proteolytic activation of the potent liver cell mitogen hepatocyte growth factor (HGF) into its active heterodimeric form [43,44], and by the recent findings that uPAR is one of the downstream targets of the HGF receptor cMet tyrosine kinase in the KM12L4 human epithelial cell line [45]. However our data did not show significant changes in the activation of cell-associated HGF during early phases of the regenerative response after CCl_4_. We also found no influence of uPAR in the ability of macrophages to populate the liver after the toxic injury. Regardless of the mechanism(s) downregulating the proliferative response, the impact of uPAR loss on hepatocyte proliferation was overshadowed by a more prominent defect in the proteolytic clearance of necrotic cells and in lobular reorganization in mice lacking uPA.

The normal liver repair in mice with the combined loss of uPAR and tPA clearly demonstrates that the presence of uPA alone (i.e., not receptor bound and without tPA-dependent activation of plasminogen) is able to restore the lobular architecture in a timely fashion. Based on the established requirement of plasminogen to implement effective liver repair, plasmin-mediated extracellular proteolysis is likely to couple receptor-free uPA to hepatic repair. Fibrin appears to be one target of plasmin in this context, but it should be noted that studies of fibrinogen-deficient mice indicate that fibrin(ogen) is not the only plasmin substrate relevant to hepatic repair [6]. Taken together, the available data suggest that soluble uPA supports hepatic repair and this appears to be achieved through a mechanism linked to plasmin-mediated proteolysis of fibrin and non-fibrin substrates at sites of injury.

## Conclusion

While binding of uPA to uPAR amplifies the properties of uPA in extra-hepatic systems, loss of uPAR did not significantly impair plasminogen activation or liver repair. The presence of its ligand uPA alone can promote plasminogen activation at levels that are sufficient to support liver repair after an acute injury.

## Competing interests

The author(s) declare that they have no competing interests.

## Authors' contributions

KS designed and performed the experiments, analyzed the data, and wrote the paper.

GES participated in experimental design and performed experiments.

JLD participated in experimental design and in writing of the paper.

JAB designed experimental strategy, analyzed the data, and wrote the paper.

All authors read and approved the final manuscript

## Pre-publication history

The pre-publication history for this paper can be accessed here:
